# Fluorescent-BOX-PCR for resolving bacterial genetic diversity, endemism and biogeography

**DOI:** 10.1186/1471-2180-8-220

**Published:** 2008-12-15

**Authors:** Lorenzo Brusetti, Iana Malkhazova, Maher Gtari, Isabella Tamagnini, Sara Borin, Maya Merabishvili, Nina Chanishvili, Diego Mora, Francesca Cappitelli, Daniele Daffonchio

**Affiliations:** 1 Dipartimento di Scienze e Tecnologie Alimentari e Microbiologiche (DISTAM), Università degli Studi di Milano, via Celoria 2, 20133, Milan, Italy; 2 G. Eliava Institute of Bacteriophage, Microbiology and Virology (IBMV) Gotua Street 3, 0160, Tbilisi, Georgia; 3 Laboratoire Microrganismes et Biomolécules Actives, Département de Biologie, Faculté des Sciences de Tunis, Campus Universitaire, 20092 Tunis, Tunisie

## Abstract

**Background:**

BOX-A1R-based repetitive extragenic palindromic-PCR (BOX-PCR) is one of the most used techniques in biogeography studies of microbial isolates. However the traditional separation of BOX-PCR patterns by agarose gel electrophoresis suffers many limitations. The aim of this research was to set up a fluorescent BOX-PCR (F-BOX-PCR) assay in which separation of PCR products is automated in a capillary electrophoresis system. F-BOX-PCR was compared with the traditional BOX-PCR using bacterial strains with different G+C content (*Bacillus cereus*; *Escherichia coli*; isolates of the family *Geodermatophilaceae*). Resolution, discriminatory power and reproducibility were evaluated by assaying different electrophoretic runs, PCR reactions and independent DNA extractions. BOX-PCR and F-BOX-PCR were compared for the analysis of 29 strains of *Modestobacter multiseptatus *isolated from three different microsites in an altered carbonatic wall from Cagliari, Italy, and 45 strains of *Streptococcus thermophilus *isolated from 34 samples of the hand-made, yogurt-like product Matsoni, collected in different locations in Georgia.

**Results:**

Fluorophore 6-FAM proved more informative than HEX and BOX-PCR both in agarose gel electrophoresis (*p *< 0.004 and *p *< 0.00003) and in capillary electrophoresis (compared only with HEX, *p *< 2 × 10^-7^). 6-FAM- and HEX-based F-BOX-PCR respectively detected up to 12.0 and 11.3 times more fragments than BOX-PCR. Replicate separations of F-BOX-PCR showed an accuracy of the size calling of ± 0.5 bp until 500 bp, constantly decreasing to ± 10 bp at 2000 bp. Cluster analysis of F-BOX-PCR profiles grouped *M. multiseptatus *strains according to the microsite of isolation and *S. thermophilus *strains according to the geographical origin of Matsoni, but resulted intermixed when a BOX-PCR dataset was used.

**Conclusion:**

F-BOX-PCR represents an improved method for addressing bacterial biogeography studies both in term of sensitivity, reproducibility and data analysis.

## Background

Typing by DNA fingerprinting is a common tool used in bacterial biogeography and epidemiology studies. Several bacterial species can be differentiated in clonal lines associated to specific animal hosts [1]. Single genetic differences between clonal lineages could be useful to determine the history of an infection or to find new possible borderline strains [2]. Similarly, fingerprinting methods are frequently used to evaluate the global dispersal of environmentally relevant microbial species or lineages in a species [3], to correlate specific genotypes to a given environmental conditions [4] and to evaluate the endemicity of a given microbial type [5,6].

Different DNA-based typing methodologies are now available and BOX-PCR is the most commonly used technique due to its simplicity, efficiency and low cost. This is a particular version of repetitive extragenic palindromic-PCR (rep-PCR) [7] that uses the BOX-A1R primer [8]. BOX-PCR is a fingerprinting analysis based on the BOX dispersed-repeat motif, firstly identified in *Streptococcus pneumoniae*, but common in a number of bacterial species [9-11]. Since the BOX repetitive sequences are interspersed throughout the genome, BOX-PCR is a method potentially capable of simultaneously surveying many DNA regions scattered in the bacterial genome. It has been shown to have similar or even better strain differentiation power, as well as to be easier to perform, than ribosomal intergenic spacer analysis (RISA), restriction fragment length polymorphism (RFLP), amplified fragment length polymorphism (AFLP), random amplified polymorphic DNA (RAPD) and other techniques [12-14]. BOX-PCR is quicker, cheaper, and in many cases more discriminatory than pulsed field gel electrophoresis (PFGE) [14], despite is generally less reproducible. BOX-PCR patterns are not affected by the culture age of the strain to be analyzed [15] and fingerprinting output can be easily analyzed by computer assisted methods [16]. These features make BOX-PCR a frequently used tool in biogeography studies in environmental microbiology [5,17-20].

The current BOX-PCR technique, in which the amplified products are separated by agarose gel electrophoresis, suffers from several limitations like poor band resolution and run standardization for comparison of the different profiles in different gels. To overcome these limitations separation of fluorescent labelled products in automated DNA sequencer can be used [21], but this interesting improvement has been applied rarely in environmental analysis and limited to machines performing separation in long polyacrylamide gels [21,22].

In this study we show that fluorescent BOX-PCR (F-BOX-PCR), in which the separation of PCR products is performed in an Abi-Prism 310 capillary electrophoresis system, is capable of resolving endemicity and the biogeographical repartition of different bacterial populations. We first assessed suitability and reproducibility of different electrophoretic runs of different F-BOX-PCR reactions prepared from independent extractions of DNA from eight bacterial strains exhibiting different G+C content. The power of F-BOX-PCR in resolving bacterial endemicity was assessed on a collection of *Modestobacter multiseptatus *strains isolated from three different microsites of an altered ancient carbonatic wall in the old city of Cagliari, Sardinia, Italy [23]. Biogeographic segregation of different bacterial populations was tested on a collection of 45 strains of *Streptococcus thermophilus *isolated from the Caucasian home-made yogurt-like product Matsoni produced in different areas of the Georgian Caucasus [24].

## Results and discussion

### Reproducibility of BOX-PCR and F-BOX-PCR in agarose gels

The reproducibility of BOX-PCR and F-BOX-PCR in agarose gels was analyzed using strains belonging to different taxa with very diverse G+C content. We chose six strains of *Geodermatophilaceae *(G+C content, about 70%) belonging the genera *Blastococcus *and *Modestobacter*, one strain of *E. coli *(50%) and one strain of *B. cereus *(35%). The six strains of *Geodermatophilaceae *were chosen on the basis of their BOX-PCR patterns in agarose gel that should cover a wide size range. Strain DS3 had a BOX-PCR pattern with a relatively small range of fragment length between 300 and 800 bp. Strains CI1-23 and CO2-33 showed BOX-PCR patterns with a wider range of fragment length, between 300 and about 2500 bp and between 300 and about 3000 bp respectively. Strains CI2-13, CI2-17 and CI2-23 were also analyzed since they had very similar patterns.

The average number of fragments found with BOX-PCR varied between 6.3 (*B. cereus*) and 16.6 (*E. coli*) with relatively high standard deviations (SD) between 0.9 and 4.0 (average SD = 2.2). Number of bands could be variable among replicates from independent DNA extractions, PCR or agarose gels: for example, *B. cereus *BOX-PCR patterns were represented by 3 to 10 fragments, strain CI2-17 pattern varied between 9 and 18 fragments, while *E. coli *pattern showed 14 to 19 bands.

**Figure 1:**
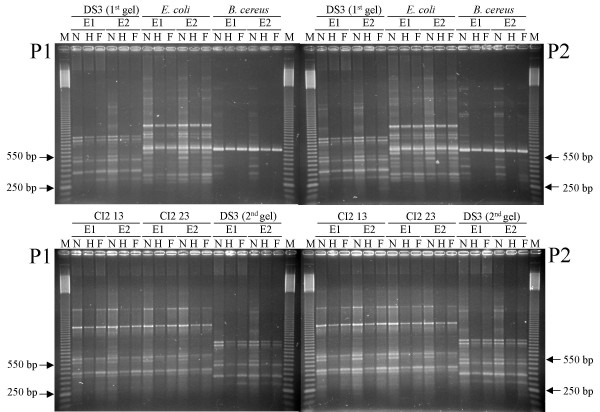
**Examples of agarose gels showing BOX-PCR obtained with the non fluorescent and fluorescent primers**. Letter N indicates the non fluorescent primer, H indicates HEX primer, while F indicates 6-FAM primer. Letter E indicates the DNA extractions performed twice (E1 and E2), while letter P indicates the PCR performed twice (P1 and P2). For strain DS3, two diverse gels are shown. M, marker 50 bp.

**Figure 2:**
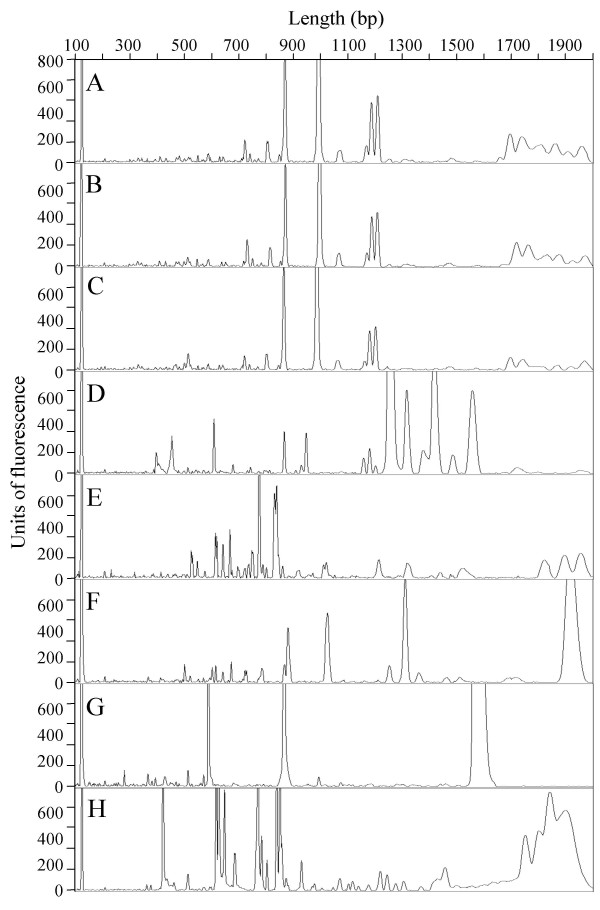
**Examples of F-BOX-PCR electropherograms after analysis with Genescan software**. Legend of letters: A. *Blastococcus *sp. CI2 13; B. *Blastococcus *sp. CI2 17; C. *Blastococcus *sp. CI2 23; D. *Blastococcus *sp. CI1 23; E. *Modestobacter *sp. CO2 33; F. *Modestobacter *sp. DS3; G. *B. cereus *360. H. *E. coli *DSM50902.

Reproducibility of BOX-PCR profiles, obtained from different DNA extractions and different runs in agarose gels, is shown in Table 1 and it is expressed as Jaccard's similarity coefficient between replicates. Examples of BOX-PCR profiles are shown in Figure 1. Reproducibility of results with standard BOX-PCR was affected by DNA extraction (86.2 ± 5.7% of similarity), PCR amplification (78.0 ± 16.1%) and gel separation (83.7 ± 9.0%). The overall similarity calculated for all replicates was of 62.7 ± 20.5%. Similarity values were occasionally quite low, as in the case of *B. cereus *which showed only 55.4% of similarities between PCR replicates and an overall similarity of 30.0%. The low reproducibility is attributable to the low number of bands for this strain. BOX-PCR patterns were seldom richer than 15–20 bands and some of these bands were weak (Figure 2). A delicate step of BOX-PCR analysis is the gel staining with ethidium bromide and UV acquisition especially of weak bands. A disappearance of one band in a profile of 10 bands decreases the similarity of two identical profiles by 10%. In the case of *B. cereus*, the average number of bands was only 6.8 and the absence of a weak band between two replicates affects the similarity by 15%.

**Table 1:** Reproducibility of F-BOX-PCR analysis with HEX and 6-FAM dyes calculated as values of Jaccard's coefficient, in comparison with standard BOX-PCR with separation in agarose gel.

Strain	Agarose gel				HEX-F-BOX				6-FAM-F-BOX			
			
	DNA extraction	PCR	Gel	Overall	DNA extraction	PCR	Injection	Overall	DNA extraction	PCR	Injection	Overall
*B. cereus *BC360	0.822	0.554	0.651	0.300	0.985	0.985	1.000	0.971	1.000	1.000	1.000	1.000
CI1-23	n.d.^a^	0.906	0.906	0.812	n.d.	n.d.	n.d.	n.d.	n.d.	0.972	0.972	0.972
CI2-13	0.864	0.952	0.952	0.818	n.d.	0.838	0.983	0.823	0.970	0.994	0.994	0.951
CI2-17	n.d.	0.624	0.811	0.500	n.d.	0.944	0.981	0.926	n.d.	0.961	0.980	0.942
CI2-23	0.907	0.905	0.864	0.727	0.961	0.983	0.994	0.933	0.969	0.969	1.000	0.939
CO2-33	n.d.	0.830	0.826	0.750	n.d.	n.d.	n.d.	n.d.	n.d.	0.978	1.000	0.978
DS3	0.791	0.597	0.803	0.375	0.971	0.971	0.990	0.923	0.919	0.979	0.992	0.950
*E. coli *DSM50902	0.927	0.874	0.879	0.737	0.979	0.964	0.993	0.943	0.919	0.977	0.992	0.882

Average	0.862	0.780	0.837	0.627	0.974	0.948	0.990	0.920	0.955	0.979	0.991	0.952
Std. Deviation	0.057	0.161	0.090	0.205	0.010	0.056	0.007	0.051	0.035	0.013	0.010	0.035

^a ^n.d. not determined

Polyacrylamide gels and silver staining gives better resolution of fragments with respect to agarose gel electrophoresis but is labour-intensive and can suffer a relatively low reproducibility due to the gel staining procedures. However it would be worth investigating the convenience of applying polyacrylamide gel electrophoresis for analysis of BOX-PCR patterns in comparison with F-BOX-PCR in terms of economic costs, time required in sample handling and overall reproducibility.

For all the strains that were tested, the use of BOX-A1R primer labelled with fluorescent chromophores affected the BOX-PCR profiles in agarose gel by decreasing the number of detectable bands (Figure 1 and Table 2); in case of *B. cereus *only one band was often visible for both HEX and 6-FAM labelled primers. Band pattern modifications were also noted in denaturing gradient gel electrophoresis (DGGE) for products labelled with 6-FAM dye in comparison with those non labelled [25]. Computer-assisted analysis of agarose gel electrophoresis showed that patterns obtained with 6-FAM labelled primer were generally richer in bands than those obtained with the fluorophore HEX (p < 0.0004). A similar result was observed by Ranjard and collaborators [26], who found 6-FAM primers giving the best total peak intensity in Automated rRNA Intergenic Spacer Analysis (ARISA).

### Reproducibility of capillary electrophoresis

The F-BOX-PCR products labelled with HEX and 6-FAM were run twice in capillary electrophoresis. For all the strains tested, peak number obtained by using 6-FAM was significantly higher than DNA band numbers in agarose gel profiles obtained by BOX-PCR (*p *< 3 × 10^-6^) and 6-FAM-based F-BOX-PCR. 6-FAM peak profiles were confirmed to be generally richer than HEX patterns (*p *< 2 × 10^-7^). The average number of peaks varied between 31.0 (strain DS3) and 72.0 (strain CI1-23) with very low relative standard deviations (between 0.0 and 2.0; Table 2). Altogether, F-BOX-PCR gave more informative patterns (1.9 to 12.0 times) than BOX-PCR, indicating that capillary electrophoresis detects at least two times more peaks than agarose gel stained with ethidium bromide. 6-FAM was more informative than HEX when comparing these F-BOX-PCRs with standard BOX-PCR (1.3 to 11.3; *p *< 3 × 10^-8^). The richness of 6-FAM-based profiles is evident in Figure 2, in which an example of run for each strain tested is shown. Profiles were well characterized by a number of high, medium and above all, small peaks that were sometimes very important. For example in the profile of *B. cereus *360 (Figure 2G) three high peaks were visible (5100 ± 1800 units of fluorescence at 601 bp, 3000 ± 1700 at 893 bp and 4800 ± 1900 at 1555 bp), while other 33 were smaller than 200 units of fluorescence. The peak patterns of strains CI2-13, CI2-17 and CI2-23 were very similar with an average number of peaks of 47.3, 50.5 and 48.3 respectively. The comparison with their profiles in agarose gel amplified with the BOX-A1R primer (Figure 1) showed how the fluorescent system could reveal much more amplicons than the traditional agarose gel.

**Table 2:** Number of fragments detected by BOX-PCR and F-BOX-PCR analysis using agarose and capillary electrophoresis separation and ratios between the number of fluorochrome-labeled fragments detected by capillary electrophoresis and the number of unlabeled fragments detected by agarose electrophoresis^a^.

Strain	DNA extraction^a^	PCR^a^	Gel/Injection^a^	Number of observed fragment					RH^b^	RF^c^
						
				On agarose gel			On AbiPrism 310			
							
				Non fluorescent	HEX	6-FAM	HEX	6-FAM		
*B. cereus *BC360	1	1	1	7	1	1	34	36	4.9	5.1
	1	1	2	8	n.d.^e^	n.d.	34	36	4.3	4.5
	1	2	1	3	1	1	34	36	11.3	12.0
	1	2	2	3	n.d.	n.d.	34	36	11.3	12.0
	2	1	1	10	1	5	34	36	3.4	3.6
	2	1	2	7	n.d.	n.d.	34	36	4.9	5.1
	2	2	1	7	1	1	33	36	4.7	5.1
	2	2	2	5	n.d.	n.d.	33	36	6.6	7.2
CI1 23	1	1	1	13	8	11	n.d.	72	n.a.	5.5
	1	1	2	13	n.d.	n.d.	n.d.	72	n.a.	5.5
	1	2	1	16	6	10	n.d.	70	n.a.	4.4
	1	2	2	13	n.d.	n.d.	n.d.	70	n.a.	5.4
CI2 13	1	1	1	9	5	6	34	41	3.8	4.6
	1	1	2	9	n.d.	n.d.	34	41	3.8	4.6
	1	2	1	10	5	7	28	41	2.8	4.1
	1	2	2	9	n.d.	n.d.	29	41	3.2	4.6
	2	1	1	11	5	6	34	40	3.1	3.6
	2	1	2	11	n.d.	n.d.	33	40	3.0	3.6
	2	2	1	10	6	7	34	39	3.4	3.9
	2	2	2	11	n.d.	n.d.	34	40	3.1	3.6
CI2 17	1	1	1	10	7	10	26	50	2.6	5.0
	1	1	2	9	n.d.	n.d.	26	49	2.9	5.4
	1	2	1	18	4	10	27	52	1.5	2.9
	1	2	2	13	n.d.	n.d.	26	51	2.0	3.9
CI2 23	1	1	1	8	4	5	44	49	5.5	6.1
	1	1	2	12	n.d.	n.d.	44	49	3.7	4.1
	1	2	1	10	6	7	44	48	4.4	4.8
	1	2	2	11	n.d.	n.d.	44	48	4.0	4.4
	2	1	1	11	4	6	45	49	4.1	4.5
	2	1	2	11	n.d.	n.d.	45	49	4.1	4.5
	2	2	1	9	4	6	43	47	4.8	5.2
	2	2	2	12	n.d.	n.d.	44	47	3.7	3.9
CO2 33	1	1	1	12	3	5	n.d.	46	n.a.	3.8
	1	1	2	10	n.d.	n.d.	n.d.	46	n.a.	4.6
	1	2	1	9	3	5	n.d.	45	n.a.	5.0
	1	2	2	11	n.d.	n.d.	n.d.	45	n.a.	4.1
DS3	1	1	1	14	6	7	35	34	2.5	2.4
	1	1	2	13	6	6	35	34	2.7	2.6
	1	2	1	7	5	7	33	34	4.7	4.9
	1	2	2	6	4	5	33	34	5.5	5.7
	2	1	1	16	6	8	34	30	2.1	1.9
	2	1	2	13	6	7	33	31	2.5	2.4
	2	2	1	13	6	7	33	32	2.5	2.5
	2	2	2	8	6	6	33	32	4.1	4.0
*E. coli *DSM50902	1	1	1	19	10	13	26	57	1.4	3.0
	1	1	2	17	n.d.	n.d.	26	57	1.5	3.4
	1	2	1	18	3	9	24	57	1.3	3.2
	1	2	2	14	n.d.	n.d.	25	57	1.8	4.1
	2	1	1	19	8	14	26	58	1.4	3.1
	2	1	2	16	n.d.	n.d.	26	57	1.6	3.6
	2	2	1	15	9	15	26	60	1.7	4.0
	2	2	2	15	n.d.	n.d.	26	60	1.7	4.0

^a ^The comparison between BOX-PCR analysis and F-BOX-PCR analysis separated in agarose gel electrophoresis or in capillary electrophoresis was done considering eight strains belonging to *B. cereus *(G+C content 35%), to the family *Geodermatophilaceae *(G+C content 60%) and to *E. coli *(G+C content 50%). The analysis was done in duplicate considering two independent DNA extractions (numbered 1, 2), two independent PCR amplifications (1, 2) and two independent agarose gel runs or sample injection in the sequencer (1, 2)

^b ^The numbers express the ratio of HEX-labeled amplicons detected by capillary electrophoresis versus unlabeled amplicons detected on agarose gels

^c ^The numbers express the ratio of 6-FAM-labeled amplicons detected by capillary electrophoresis versus unlabeled amplicons detected on agarose gels

n.d. not determined.

n.a. not applicable.

Since run characteristics along a capillary are nonlinear and most commercial size standards can present double peaks, there are greater uncertainties in sizing large DNA fragments. The Local Southern sizing method of Genescan software may improve the fragment sizing, generating the best-fit sizing curve. The software gives size in base pairs as a function of migration time of standard fragments. In our replicates we observed that up to 500 bp the precision of separation in all samples was close to ± 0.5 bp, but over that, size precision constantly decreased reaching ± 1 bp at 800 bp, ± 5 bp at 1400 and ± 10 bp at 2000 bp. Over 1500 bp, the capability of separation of the 47 cm-capillary provided with POP4 polymer is reduced heavily appreciably affecting the sizing process with peak size discrepancy of ± 20 bp between replicated samples. Over 1300 bp, base peaks became wider and tended to merge as it can be noted in *E. coli *(Figure 2H). However, the precision of the region between 1300 and 2000 bp in perfect conditions of run can be acceptable in comparison with agarose or polyacrylamide gels, since the seven peaks at 1700–2000 bp of the similar strains CI2-13, CI2-17 and CI2-23 can be easily aligned manually (Figure 2A, 2B, 2C). The problem of the discrepancy in sizing large fragments is reflected in the set up of the data matrix due to the size calling precision (± 0.01 bp). This uncertainty may cause F-BOX-PCR to appear less similar to each other, caused by splitting of peaks between adjacent combination windows [27]. Sizing interpolation of large peaks beyond 1000 bp could be improved by an improvement of polymers used in fragment separation or in innovative downstream matrix analysis. In spite of these uncertainties, the overall percentage of similarity of replicated analyses for 6-FAM-based F-BOX-PCR was 95.2 ± 3.5% (Table 1). As observed for agarose gels, the reproducibility of the technique is mainly affected by different DNA extractions and by different PCR amplifications than by capillary separation (Table 1).

### F-BOX-PCR typing of *M. multiseptatus *from different stone microsites

We used F-BOX-PCR for analyzing bacterial endemisms on the surface of three microsites of a highly biodeteriored calcarenite stone characterized by a relatively high rate of erosion due to rainwater, saltiness and wind. We previously found that this stone was heavily colonized by actinobacteria of the family *Geodermatophilaceae*, but typing with standard BOX-PCR failed to find clear relation between isolates and the microsites where they came from [23] despite these microsites were characterized by different colours and types of alterations (pittings, patinas, etc.).

**Figure 3:**
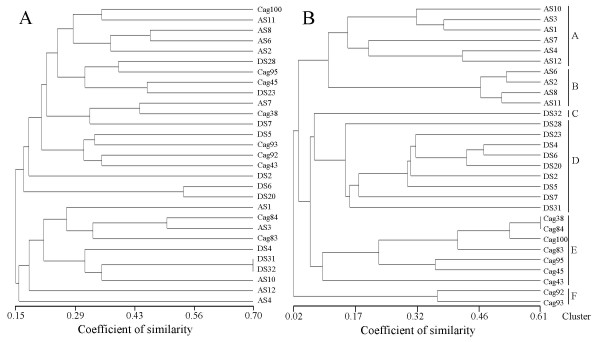
**Similarity UPGMA trees of BOX-PCR patterns (A) and F-BOX-PCR patterns (B) of 29 bacterial strains belonging to *Modestobacter *sp. isolated from three different microsites of an altered carbonatic stone wall in the old city of Cagliari**. The UPGMA tree originated by F-BOX-PCR was divided into 6 clusters on the basis of Pearson's r correlation value.

We compared F-BOX-PCR with BOX-PCR, by analyzing with the two methods, 29 strains of *M. multiseptatus *isolated from the surface of three different microsites on the stones (microsites A, C and D). The number of bands detected by BOX-PCR were between 5 (strains AS4 and AS10) and 17 (strain Cag100) with an average of 10.3. Band size was between 250 and 2500 bp. UPGMA tree failed to clearly cluster strains on the basis of the microsite of isolation (Figure 3A). The similarity of groups was between 15 and 70%. The comparison between the cophenetic similarity matrix and the original similarity matrix done with the Mantel's test gave a product-moment correlation of 0.68, a very low fit value that do not make UPGMA output significant. F-BOX-PCR of these strains gave between 25 (AS12) and 49 (DS32) peaks with an average of 37.1. Peak size was between 150 and 1978 bp. The average height of the peaks was 310 units of fluorescence. Strains were grouped in the UPGMA tree according to the microsite of isolation (Figure 3B). Strains isolated from microsite A clustered in groups A and B, with an overall similarity of 11%. Strains from microsite C were segregated in cluster E with most of the strains with a similarity of 9% and cluster F including strains Cag92 and Cag93 with a similarity of 38%. Strains of microsite D clustered in one group (D) with 14% similarity, while strain DS32, although in relation with cluster D, appeared to be separated. The coefficients of similarity were lower than the BOX-PCR tree due to the number of peaks, 3.6 times higher than bands in the agarose gels. A very high product-moment correlation (0.96) characterized F-BOX-PCR tree. Since clusters of Figure 3B were constituted by strains of homogeneous microsite origin, Pearson's correlation between sample location and genomic clusters shown in UPGMA tree was 1.00.

### F-BOX-PCR typing of *S. thermophilus *isolates from different source in Georgia

For evaluating if F-BOX-PCR can discriminate bacterial population on a geographical scale, the bacterial diversity of 45 strains of *S. thermophilus *isolated from the Georgian yoghurt Matsoni [24] produced in 34 different farms was studied (Table 3).

**Table 3:** Geographical origin and F-BOX-PCR cluster of *Streptococcus thermophilus *strains isolated from Matsoni.

Strain	Sampling place	Longitude	Farm	Geographical area^1^	F-BOX-PCR cluster
3252	Batumi	41° 38' E	21	Black Sea (*S*)	C2
3238	Gantiadi	41° 43' E	17	Black Sea (*S*)	C1
3240	Gantiadi	41° 43' E	18	Black Sea (*S*)	C1
3242	Gantiadi	41° 43' E	18	Black Sea (*S*)	C1
3232	Kobuleti	41° 47' E	14	Black Sea (*S*)	C1
3233	Kobuleti	41° 47' E	14	Black Sea (*S*)	C1
3248	Perva	41° 48' E	20	Black Sea (*S*)	E
3203	Mtskaldidi	41° 48' E	1	Black Sea (*S*)	A
3245	Oryabatumi	41° 48' E	19	Black Sea (*S*)	C1
3202	Senaki	42° 03' E	16	West Georgia (*W*)	A
3235	Senaki	42° 03' E	15	West Georgia (*W*)	C1
3236	Senaki	42° 03' E	15	West Georgia (*W*)	C1
3217	Kvitiri	42° 37' E	10	West Georgia (*W*)	B
3219	Kvitiri	42° 37' E	10	West Georgia (*W*)	F
3206	Kutaisi	42° 42' E	6	West Georgia (*W*)	A
3207	Kutaisi	42° 42' E	6	West Georgia (*W*)	A
3213	Kutaisi	42° 42' E	9	West Georgia (*W*)	B
3221	Godogani	42° 46' E	11	West Georgia (*W*)	B
3222	Godogani	42° 46' E	11	West Georgia (*W*)	B
3225	Godogani	42° 46' E	12	West Georgia (*W*)	C
11A	Zestaphoni	43° 00' E	33	Mountain area (*M*)	D
12A	Zestaphoni	43° 00' E	34	Mountain area (*M*)	D
4B	Bakuriani	43° 31' E	38	Mountain area (*M*)	D
4A	Bakuriani	43° 31' E	37	Mountain area (*M*)	D
3211	Surami	43° 33' E	8	Mountain area (*M*)	F
3212	Surami	43° 33' E	8	Mountain area (*M*)	F
1D	Khashuri	43° 35' E	36	Mountain area (*M*)	D
10B	Metekhi	44° 20' E	42	Mountain area (*M*)	D
10C	Metekhi	44° 20' E	42	Mountain area (*M*)	D
3261	Tskhneti	44° 38' E	25	Tbilisi area (*T*)	E
3B	Tskhneti	44° 38' E	39	Tbilisi area (*T*)	D
1720	Mtskheta	44° 42' E	44	Tbilisi area (*T*)	D
3263	Tabakhmela	44° 44' E	26	Tbilisi area (*T*)	C2
3265	Tabakhmela	44° 44' E	27	Tbilisi area (*T*)	C2
3266	Tabakhmela	44° 44' E	27	Tbilisi area (*T*)	C2
3270	Shiheligi	44° 46' E	28	Tbilisi area (*T*)	C2
3271	Shiheligi	44° 46' E	28	Tbilisi area (*T*)	C
3273	Teleti	44° 51' E	29	Tbilisi area (*T*)	C2
3275	Teleti	44° 51' E	30	Tbilisi area (*T*)	C2
3276	Teleti	44° 51' E	30	Tbilisi area (*T*)	C2
3278	Teleti	44° 51' E	31	Tbilisi area (*T*)	E
3279	Teleti	44° 51' E	32	Tbilisi area (*T*)	D
5B	Tsxvarichamia	44° 55' E	40	Mountain area (*M*)	D
6A	Gombori	45° 14' E	41	Mountain area (*M*)	D

^1 ^Four geographical areas were defined according to longitude and altitude of the sampling places. The Black Sea area (*S*) includes farms located on the coast between longitudes 41°00 E and 42°00 E. The West Georgia area (*W*) includes farms between longitudes 42°00 E and 43°00 E. The Tbilisi area (*T*) includes farms between 44°00 E and 45°00 E. The mountain area (*M*) includes farms in a wide range of longitudes (from 43°00 E to 45°14 E), which having in common an altitude higher than 800 m.

**Figure 4:**
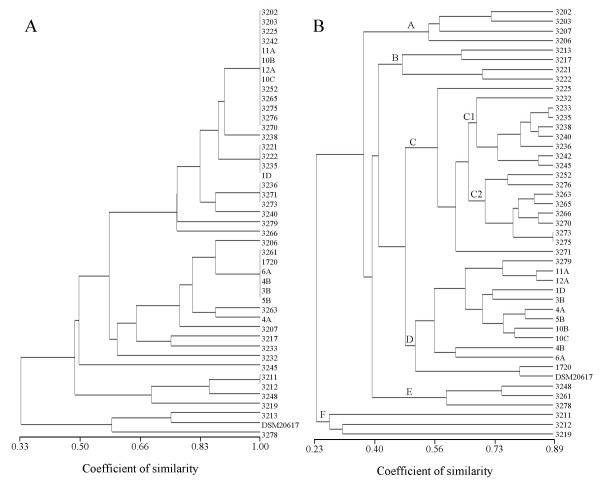
**Similarity UPGMA trees of BOX-PCR patterns (A) and F-BOX-PCR patterns (B) of 45 bacterial strains belonging to *S. thermophilus *isolated from the typical yoghurt-like product Matsoni produced in 34 different farms in Georgia**. The UPGMA tree originated by F-BOX-PCR described 6 clusters (A to F) and 2 subclusters (C1 and C2), on the basis of Pearson's r correlation value.

The number of bands detected by BOX-PCR were between 2 (for strain 3278) and 10 (strain 3238) with an average of 7.5 bands and size was between 250 and 2000 bp. Although some homogeneous groups were detectable in UPGMA tree (Figure 4A), cluster analysis failed to clearly group strains on the basis of the geographical origin of Matsoni (r = 0.59, *P *= 2 × 10^-5^). The similarity of groups was between 33 and 100%. Five clusters included strains with identical BOX-PCR patterns, although strains were isolated from different regions in Georgia. Comparison between the cophenetic similarity matrix and the original similarity matrix done with the Mantel's test gave a product-moment correlation of 0.94, a very high fit value related to the relative low number of bands in the agarose gel and the high number of identical patterns.

F-BOX-PCR of the same strains gave between 11 (strain 3211) and 28 (strains 3232 and 3276) peaks with an average of 22.2 and sizes between 119 and 539 bp. The average peak height was 285 fluorescence units. The similarity coefficients were lower than the BOX-PCR tree (from 23 to 89%) as observed with the previous analysis of *M. multiseptatus*, but it is explained by the number of peaks three times higher than the bands in the agarose gel. F-BOX-PCR did not produce identical patterns among the strains, hence the diversity in the peak matrix increased. For this reason, although the product-moment correlation of F-BOX-PCR tree was lower than in the agarose gel-derived UPGMA tree (0.88 vs. 0.94), the fit of the analysis is still positive.

**Figure 5:**
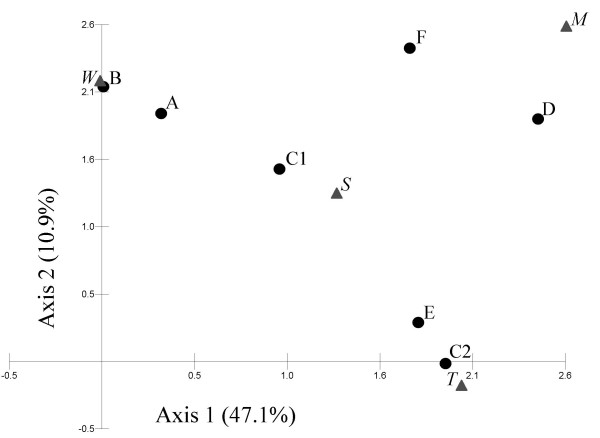
**Detrended Principal Coordinate Analysis plot showing the relationships between the UPGMA groups defined by F-BOX-PCR and the ecotypes determined from the geographical characteristics of sampling sites**. Letters (A to F) indicate the six F-BOX-PCR profile clusters as reported in Figure 4. Letters in italic indicated the geographical characteristics of the sampling sites, according to Table 3 (*M*, mountain area; *S*, Blak sea; *T*, Tbilisi area; *W*, West Georgia).

F-BOX-PCR clustered *S. thermophilus *strains in the UPGMA dendrogram according to the geographical origin of the product from which they were isolated (r = 0.81, *P *= 3 × 10^-11^). Most of the strains isolated from western Georgia were clustered in two groups (A and B in Figure 4B) with similarity of 54% (including strains collected between 41° 48' E and 42° 42' E) and 46% (strains isolated between 42° 37' E and 42° 46' E). Within group A, it is placed strain 3203 from Mtskaldidi, a place relatively close to Senaki area. Cluster C includes with a similarity of 56% two subclusters (C1 and C2) with an average similarity of about 70%. Strains from the Black Sea coast (between 41° 43' E and 41° 48' E) were grouped into C1, with two strains isolated from inner western Georgia (3235 and 3236, both from Senaki, 42° 03' E, close to the Black Sea coast). Subcluster C2 was mainly formed by strains isolated in the surrounding area of Tbilisi (between 44° 44' E and 44° 51' E), plus strain 3252 isolated in Batumi. Cluster D was formed by strains collected from various farms located in the mountain area of Central Georgia. The remaining strains were grouped in two groups (E and F) characterized by an overall homogeneous geographical origin, although group F had a low strain similarity (26%). Detrended Principal Coordinate Analysis was used to cluster UPGMA groups defined by F-BOX-PCR with ecotypes determined from the geographical characteristics of sampling site (Figure 5). The resulting plot, representing the 58.0% of the total inertia, confirmed the correlation found in the UPGMA tree. For example, clusters C2 and E were close to the Tbilisi area (*T*), as expected, since they were mostly formed by strains isolated from that region. Strong relationships were also found for groups A and B according to the western origin of their samples (*W*), for C1 including strains originating from the Black Sea coast (*S*) and, partially, for D, including isolates from the mountain area (*M*).

## Conclusion

Our study showed that a bacterial DNA fingerprinting technique that use automated procedures for DNA fragment separation could be an advantageous methodology to type and track microbial isolates and define endemisms in homogeneous environments where differential pressure is exerted by secondary (minor) environmental factors. In the case of *M. multiseptatus*, while standard BOX-PCR failed to find a clear strain clustering on the basis of sampling site [23], F-BOX-PCR gave a very well defined grouping with a clear relation with the microsite of isolation. This indicated that the technique can highlight endemisms in epilytic/endolytic environments, despite the environmental factors driving this endemism remain unknown and are still to be explored. In case of *S. thermophilus*, our data showed that F-BOX-PCR succeeded in defining a relation of strain types with the area of isolation, evidencing a geographical specificity. Despite the relative easiness, automated fingerprinting analysis is not yet much used in the industrial system, although the final output can be analyzed by rapid, efficient and computerized analyses. F-BOX-PCR can prove as an advantageous tool to routinely depict microbial communities, for instance, for typing isolates in food industry or in traditional and regional [28,29] dairy products. F-BOX-PCR, coupled with other well-known automated techniques, could help in labelling food products with the P.D.O. (Protected Designation of Origin) and monitoring the related production chain. For example, F-BOX-PCR could be applied in parallel to Length Heterogeneity-PCR that was recently proposed as a rapid and precise method to characterise the lactic acid bacteria present in natural whey starters for the P.D.O. Grana Padano cheese and in maize silage [30,31].

## Methods

### Environmental samples and pure strains

Bacterial strains used in this work belong to *M. multiseptatus *and to *S. thermophilus *species on the basis of 16S rRNA gene analysis and species-specific PCR analysis targeted to *lacZ *gene [32] respectively. All strains isolated from Matsoni were also tested by carbohydrate fermentation profiles (API-20 Strep, Bio-Merieux). Strains belonging to *M. multiseptatus *were isolated from an ancient carbonatic wall of the city of Cagliari (Microsite C, Sardinia, Italy) [23]. The genotypes of these isolates attributed to the family *Geodermatophilaceae *have been previously studied by BOX-PCR [23]. In addition to these strains, other *Geodermatophilaceae *were isolated from two other microsites (A and D) of the same ancient wall, as previously described [23].

From 34 samples of home-made Matsoni, 45 strains belonging to *S. thermophilus *were isolated (Table 3). Matsoni samples were collected in local market or families in different cities or villages, from the eastern to the western part of Georgia. One ml of the sample was resuspended in 9 ml sterilized 0.85% NaCl solution and mixed thoroughly. Serial dilutions (10^-1 ^to 10^-8^) were prepared and 0.1 ml of appropriate dilution was spread in duplicate onto M17 agar plates. After incubation (37°C, 24–48 h, aerobic conditions) strains were purified by streak plating and cultivated in M17 broth using lactose at the final concentration of 2% (w/v). Stock cultures were stored in glycerol solution (20%) at -80°C.

*Modestobacter *sp. DS3 and CO2-33, *Blastococcus *sp.CI1-23, CI2 17, CI2-13 and CI2-23, *Escherichia coli *DSM50902 and *Bacillus cereus *360 [17] were used as reference strains to test reproducibility of F-BOX-PCR.

### BOX-PCR and F-BOX-PCR

DNA was extracted from the bacterial cultures as described elsewhere [33]. Genomic DNA integrity was checked by agarose gel electrophoresis and quantification was measured with a SmartSpec 3000 spectrophotometer (Biorad, Milan, Italy). BOX-PCR was performed by using the BOX-A1R primer [8] as previously reported [23] with 15 ng of the DNA as template. The PCR product was run in agarose gels electrophoresis and banding patterns were acquired from the ethidium-bromide stained gels with GelDoc 2000 image system (Biorad) and stored on disk as 1sc files. The "rolling disk" background subtraction method was applied to each gel and a database containing all the gel images was created. Bands were automatically detected and normalized using the 50 bp DNA ladder (Pharmacia) as the molecular size marker. F-BOX-PCR was performed following the method of Urzì and collaborators [23] with the following modifications. Mixtures contained 1× PCR buffer (Pharmacia, Milan, Italy), 2 mM MgCl_2_, 0.1 mM dNTPs, 0.8 μM of each fluorescent primer, 5% of dimethylsulfoxide, 1.3 U of *Taq *DNA polymerase (Pharmacia) and 15 ng of genomic DNA in a final volume of 30 μl. Primer BOX-A1R was labelled alternatively with 6-FAM (6-carboxyfluorescein) or HEX (6-carboxyhexafluorescein) at their 5' end. Reactions were denatured at 94°C for 5 min, subjected to 35 cycles of 94°C for 1 min, 45°C for 1 min and 72°C for 2 min. A final extension at 72°C for 10 min was added. The amount of PCR products were estimated on agarose gel and 3 μl of each product were added to 15 μl of deionized formamide, containing 1 μl of 2500 ROX-labelled internal size standard (Applied Biosystems, Monza, Italy). Samples were denatured at 95°C for 10 min and rapidly put into ice for 7 min. F-BOX-PCR fragments were loaded on a 310 Abi Prism capillary electrophoresis in denaturing conditions using POP4 running polymer and a 47 cm × 50 μm capillary. Samples were run for 60 min at 15 kV. The injection time of each sample was 5 sec at 15 kV. Data were analyzed with Genescan 3.1.2 software (Applied Biosystems): a threshold of 50 fluorescent units was used, corresponding to 2 times the highest peak value detected during the negative control run, and sizing was done with the Local Southern Method and light data smoothing. If the baseline varied inconsistently, the sample was rerun.

### Reproducibility of F-BOX-PCR

Genomic DNA was extracted by two different operators and the subsequent PCRs with the agarose gel runs were executed by two different operators as well. Each PCR was loaded twice in the sequencer. Each sample loaded was subjected to two separate injections. Duplicate injections of each sample of the Abi Prism 310 were analyzed for replicated peaks. Peaks present in only one duplicate were discarded, since they probably are salt spikes of the POP4 running polymer (Genescan Reference Guide). Moreover, peaks shorter than 3000 data points were not analyzed, since they are formed by fluorescent primers and primer dimers.

### Statistical analysis

Average number of bands or peaks and standard deviation were performed. Comparisons between band or peak number and between efficiency values of each technique were performed with the Student's t test. The band and peak matrices corresponding to the BOX-PCR and F-BOX-PCR profiles were subjected to a cluster analysis. A binary 0/1 matrix was created basing on the absence or presence of DNA bands or peaks. Pairwise distances were calculated with the SimQual function of the NTSYSpc 2.01 computer program (Applied Biostatistics Inc., USA) by employing the Jaccard's coefficient for two-state data and strain clustering was performed by the UPGMA analysis [34]. The significance of the resulting tree was checked by comparing the original similarity matrix with the cophenetic similarity matrix by using the Mantel test. The correlation between the geographical origin of Matsoni from which the strains were isolated and the subclusters which were identified by the UPGMA analysis, was done with both the Pearson's r correlation test using the PAST software [35] and a Detrended Principal Coordinate Analysis using the MVSP 3.13n software (Kovach Computing Services, Anglesey, UK). Data scores were detrended and no species weighting was applied. The number of axes to be extracted was calculated by Gittman-Kaiser's familiar eigenvalue rule [36].

## Authors' contributions

LB and DD conceived the study design, coordinated the overall work and participated in writing the manuscript. LB: carried out all the statistical analyses, and participated in molecular biology experiments. MG: carried out isolation and identification of bacterial strains from stone surface. DM: carried out identification of *S. thermophilus *strains. IT: carried out BOX-PCR and F-BOX-PCR setup experiments and participated in writing the manuscript. IM: carried out F-BOX-PCR experiments on *S. thermophilus *strains. SB, NC, FC and MM participated in BOX-PCR and F-BOX-PCR of the bacterial isolates. All authors read and approved the final manuscript.
